# Preferences and Experiences of Parents/Guardians of Adolescents and Young Adults with Epilepsy and Intellectual Disability Regarding Decision-Making Surrounding Contraception

**DOI:** 10.1016/j.jpag.2025.05.004

**Published:** 2025-05-25

**Authors:** Geetha Vasudevan, Robyn Filipink, Jenna Gaesser, Traci M. Kazmerski, Yoshimi Sogawa, Laura Kirkpatrick

**Affiliations:** 1 UPMC Children’s Hospital of Pittsburgh, Pittsburgh, Pennsylvania; 2 Division of Adolescent and Young Adult Medicine, UPMC University Center, Pittsburgh, Pennsylvania; 3 Center for Innovative Research on Gender Health Equity, Pittsburgh, Pennsylvania

**Keywords:** Sexual health, Reproductive health care, Epilepsy, Intellectual disability, Contraception

## Abstract

**Objective:**

To explore the preferences of parents/guardians of adolescents and young adults (AYAs) of childbearing potential with co-occurring epilepsy and intellectual disability (ID) regarding decision-making on contraception

**Methods:**

We conducted semi-structured interviews with parents/guardians of female AYAs (12–28 years old) with co-occurring epilepsy and ID recruited from a tertiary-care children’s hospital. We confirmed the diagnoses of epilepsy and ID with the patient’s neurologist and parent/guardian. All degrees of ID (eg, mild/moderate/severe) were eligible. We audio-recorded and transcribed interviews. Two coders performed qualitative thematic analysis.

**Results:**

Twenty-five parents/guardians completed the interviews. Themes included the following: (1) Parents’/caregivers’ desire for birth control for their child was more centered around menstruation-related concerns rather than pregnancy prevention; (2) many parents were afraid of potential adverse effects of contraception for their children, including perceptions that risks might outweigh benefits; (3) a common important factor in contraceptive decision-making was ease of administration, and, generally, the pill form was preferred; (4) longer-acting methods, if chosen, were largely selected due to trusting recommendations from health care providers yet were often feared due to perceptions of lower ease of reversibility if problems arise, as well as greater challenges in delivery or placement.

**Conclusion:**

Findings may inform interventions to improve contraceptive care for AYAs with epilepsy and ID, including development of discussion guides and decision aids for parents/caregivers.

## Introduction

Epilepsy and intellectual disability (ID) are diagnoses that co-occur at a rate of approximately 25%, with the rate of epilepsy rising with increasing severity of ID.^1,2^ Adolescents and young adults (AYAs) with epilepsy have specific considerations regarding starting contraception, including drug interactions between antiseizure medications and contraceptives. An added layer of complexity occurs when parents/guardians are the decision-makers for AYAs with epilepsy and ID due to the AYAs potentially not having the capacity to make independent medical decisions. In all young patients, there is a need for education on emerging sexuality, and parents of young persons with ID may experience additional challenges with supporting menstrual care and have concerns regarding the risk of sexual abuse, the risk of sexually transmitted infections, and the optimization of managing fertility.^4–14^ The American College of Obstetricians and Gynecologists recommends ideally starting contraception-related conversations early in adolescence and routinely revisiting the topic on the basis of changing contexts. The American College of Obstetricians and Gynecologists encourages collaboration with the patient’s neurologist given overlapping topics of importance, such as drug-drug interactions, teratogenicity, and catamenial seizures.^3,14^ To best center contraceptive counseling in patient and family values, clinicians must further understand parent/guardian perspectives about initiating contraception and selecting a method. There is little information in the literature about the perspectives of parents/guardians in making contraceptive decisions, especially for those with co-occurring epilepsy and ID.^3–14^

This study used a qualitative methodology to explore parent and guardian preferences for and experiences with decision-making surrounding initiation of contraception and selection of a method for AYAs with co-occurring epilepsy and ID. The findings can inform providers on how to improve sexual and reproductive health (SRH) care and facilitate patient- and guardian-oriented discussions about contraception for AYAs with co-occurring ID and epilepsy.

## Methods

We used a qualitative methodology to explore parental/guardian preferences and experiences regarding decision-making surrounding contraception for their AYAs with co-occurring epilepsy and ID.

We recruited parents/guardians of AYAs with co-occurring epilepsy and ID. Patients were diagnosed with epilepsy before the age of 18 and could have any degree of ID (mild/moderate/severe). We confirmed the diagnoses of epilepsy and ID with the patient’s parent/guardian and primary neurologist. We recruited participants during clinic visits with their child’s pediatric neurologists in a large academic medical center in Western Pennsylvania. We interviewed patients to understand experiences around SRH, puberty, and contraception. We excluded parents/guardians with insuﬃcient English language proficiency to complete a telephone interview. We recruited and interviewed specifically the parent/guardian who accompanied their child to their child’s medical appointment. Participants received a $25 gift card as compensation.

We read the parent/guardian an informed consent script and obtained verbal informed consent before the interview. We obtained consent/assent from the AYA if they were cognitively able to participate in the consent/assent process; we asked them if they would consent/assent to their parent/guardian discussing their medical information in the interview. Interviews lasted between 20 and 60 minutes. We audio-recorded and transcribed interviews verbatim with identifiable information redacted. We used a semi-structured interview guide that was in line with standard qualitative interviewing techniques.^15^ The interviewer presented questions to the participant, then, after allowing the participant to respond, proceeded with prompts/probes to elicit further information and clarification from the participant until no further information was forthcoming. We developed the guide on the basis of previous qualitative research with pediatric neurology providers and females with epilepsy.^5,8,10^ One female pediatric neurologist (LK) performed the interviews. At the time of the interviews, she was a female pediatric neurologist and epilepsy fellow with training in qualitative methodology.

We used Braun and Clarke’s thematic analysis methodology to code transcripts and identify themes.^15^ The principal investigator (LK) created a preliminary codebook based on the interview guide, then 2 independent coders (GV, LK) reviewed and coded all transcripts. GV, at the time of coding, was a female pediatric neurology resident with training in qualitative methodology. The coders used the preliminary codebook deductively and inductively added further codes as needed. The coders convened periodically throughout the study to discuss and reconcile their coding, resolve discrepancies, and establish new codes using the consensus coding approach. They then used the final codebook to analyze the transcripts, evaluate themes, and select representative quotations. The University of Pittsburgh Institutional Review Board approved this study.

## Results

Twenty-five parents/guardians participated in the interviews. Their children’s ages ranged from 12 to 28 years old (median age 18 years), and the parent/guardian age ranged from 32 to 74 years old (median age 50 years). Table 1 describes participant demographic and epilepsy characteristics. Multiple themes emerged related parents/guardians making decisions surrounding contraception for their child. Table 2 lists quotations related to the themes.

### Theme 1. *Parents’/caregivers’ desire for contraception for their child was more centered around menstruation-related concerns rather than pregnancy prevention.*

Overall, parents/guardians wanted to initiate contraception to help with menstrual concerns rather than as a method for pregnancy prevention. They were focused on addressing issues such as hygiene, mood changes, excessive bleeding, pain, and catamenial seizures. One parent mentioned, “She did have a major increase in seizures [with her menses]. She did have pain, so we ended up putting her on Depo-Provera” when discussing their reasoning behind starting contraception. Parents perceived these issues related to menstruation as affecting their child’s quality of life. One parent said, “It is horrible to watch her being [in] such pain and miserable for four days, you know, even prior to it [her period], she was kind of moody and not feeling well.” Parents also brought up that it was hard for their AYAs to communicate what premenstrual syndrome symptoms they were experiencing, which would often lead to them guessing and trying to manage discomfort with as-needed medications to the best of their abilities.

One parent said, “It was to prevent her from having to deal with the things that come along with the period, because we didn’t know, what was going on and what she couldn’t [tolerate]. So it was supposed to be just to avoid periods altogether, just for her daily…activities.”

Parents believed that sexual activity and pregnancy were not in their child’s future, so they were less concerned about pregnancy prevention. A parent was asked about whether they imagined their AYA becoming pregnant, and they responded, “She does not got the cognitive function to be even mindful of that [sexual activity]. So at this point, that kind of information just doesn’t seem particularly relevant.”

When responses were stratified by age of the parent/guardian, those who were between 40 and 50 years old were more interested in contraception secondary to menstruation-related concerns rather than pregnancy-related concerns when compared with parents of other age groups. The other age groups were either ambivalent or did not have clear opinions on the matter.

### Theme 2. *Many parents were afraid of potential adverse effects of contraception for their children, including perceptions that risks might outweigh benefits.*

Parents/guardians who were interested in starting contraception for their AYAs began to discuss the pros and cons with various providers, including their child’s neurologist, adolescent medicine specialist, obstetrician-gynecologist, or primary care provider. Parents expressed hesitation based on potential adverse effects of contraception. Parents were particularly concerned about effects of contraception on bone density, especially given that some antiseizure medications can also cause decreased bone mineral density and some of their children had limited mobility. One parent said, “Because, of course, you already have some instability, muscle control and things like that. I didn’t know if that would affect that even more. I was told that she could take some calcium pills, but it still concerns me, right?” A few parents mentioned weight gain as one of their concerns about starting contraception. These parents identified as the primary caregiver for their child, carrying them to the bathroom and into and out of bed. They worry that weight gain beyond a safe and manageable amount could compromise their ability to care for their child. One parent said, “But I don’t want her to be obese…. Because I still carry her.”

Another common hesitation that was brought up by parents was the idea of adding more medications for a child who is already medically complex. Parents also worried about how contraception would interact with their current antiseizure medication regimen and were wary that antiseizure medication serum concentrations may need to be checked more often and that the seizure frequency may initially change as medications are optimally adjusted. One parent mentioned, “So the only thing it worries me about that, like trying another medicine… if she’s going to get that reaction [reference to a previous drug interaction with antiseizure medication experienced by her child]. That’s what scares me.”

Some parents expressed that given the risk of these adverse effects, they are willing to forgo contraception and address the symptoms of menstruation and/or catamenial epilepsy in other ways or simply not provide treatment for these symptoms. One parent stated, “You know, [her menses] may increase the seizure activity some for a few days, but nothing that – I think the risks outweigh the benefit for her at this time.”

There were no differences between parents/guardians on the basis of age regarding the adverse effects of contraception. Parents were unanimously concerned about a variety of adverse effects. Weight gain concerns were brought up specifically by parents/guardians in the age groups of 40–50 years old and 50–60 years old.

### Theme 3. *A common important factor in contraceptive decision-making was ease of administration, and, generally, pills were preferred.*

When discussing the major contributing factors parents/guardians considered when selecting a form of contraception for their child, the ease of administration was a recurring theme. For some parents, the medroxyprogesterone acetate injection was a popular option because of the infrequent and quick nature of administration occurring by injection every 3 months. One parent mentioned, “Yeah, I want to do as little as possible, but still do what we have to do.” Some parents mentioned wearing-off effects and an increase in seizures at Weeks 10–12 following the injection, requiring a change in the frequency of injections or addition of a hormonal pill for symptom management. Seeking an optimal regimen, potentially requiring more visits to the medical provider, initially caused some parental frustration, but once an optimal schedule was reached, parents felt more comfortable. However, some parents/guardians were hesitant about medroxyprogesterone acetate injections because of their child’s fear of needles and causing further medical trauma in children who are already heavily involved in the medical system.

Parents were also concerned about the diﬃculty of inserting an intrauterine device. one parent described their child undergoing severe anxiety as they headed to the appointment: “[She] went crazy in the elevator to that appointment” and required sedation to arrive in the clinic. Further sedation was required for the actual placement of the intrauterine device.

Overall, the most popular form of contraception in our sample was the oral contraceptive pill. Multiple parents mentioned the ease of administration as the leading reason for this preference. When a parent was asked about the reasoning behind choosing the pill over the medroxyprogesterone acetate injection, they said, “I figured the pill would just be the easiest.” Another parent mentioned that their child has a gastrostomy tube, which they use for medication administration, so the oral contraceptive was an easy addition to their routine: “And that was, you know, one thing that I could just give it easily.” Another parent mentioned the ease of prescription refill as well, saying, “I just go back once a year so they could just give me another year’s worth of pills.” Parents stated that the ease of administration of pills helped reduce the medical burden of their child’s experiences.

Ease of administration of the contraception was a determining factor across all parent/guardian age groups.

### Theme 4. *Longer-acting methods, if chosen, were largely selected due to trusting recommendations from health care providers yet were often feared due to perceptions of lower ease of reversibility if problems arise.*

Parents expressed concerns about ease of reversibility of the medroxyprogesterone acetate injection. They were afraid that if their child had an adverse reaction to the injection, they would be unable to discontinue the medication easily in comparison with a pill. One parent said, “Our fear was that once she has the shot, obviously, if something would not go right. You know, you can’t reverse that. And then it’s done. You know, you can’t change.” Similarly, parents were worried about identifying problems or adverse effects with the intrauterine device and the diﬃculty of the process of removing an intrauterine device if needed. As one parent said, “My concern was if there was something wrong down there, she really couldn’t tell me.”

Despite hesitations, some parents/guardians often pursued longer-acting forms of birth control after discussions with their providers. For example, one parent said, “[The doctor] gave me pros and cons of everything and her experience with children with epilepsy. She’s always found that ….the Depo is the one that seems to work the best. I just took her advice because she’s educated, she knows— she’s been there, she’s seen it. And that was important too.” Some parents expressed that they appreciated long-acting methods because of their longevity.

When responses were stratified by age of the parent/guardian, those who were between 40 and 50 years old and 50–60 years old brought up starting a long-acting reversible contraceptive for their child secondary to the trusted advice of their provider.

## Discussion

In our qualitative study of parents/guardians of AYAs with co-occurring epilepsy and ID, we observed complexity surrounding decision-making about contraception. Participants identified multiple concerns about contraceptives, including reversibility, ease of administration, adverse effects, interactions with antiseizure medications, and frequency of required medical visits. They identified goals and benefits of contraception, including management of menstrual symptoms and catamenial seizures. Understanding parent/guardian goals of treatment, preferences, and concerns surrounding contraception is imperative for promoting shared decision-making about contraception between clinicians and families, as well as empowering parents/guardians to make high-quality decisions about contraception for their child (in situations when the child is not the primary decision-maker due to lacking independent decision-making capacity).

There is limited research on how parent and guardians make decisions about contraception for people with ID who lack independent medical decision-making capacity. Previous literature has suggested that parents and guardians have a generally more cautious attitude toward contraception, mainly in the context of assessing and discussing the readiness of their child for sexual relationships, along with concern about sexual abuse.^2,16–17^ However, in our study, parents/guardians conceived of contraception as more for management of menstrual symptoms and catamenial seizures than related to pregnancy prevention. In addition, our study delved deeper into the nuances of selecting specific types of birth control once the decision for initiation of contraception has occurred compared with previous literature. More research is needed on how to optimally support patients and families in decision-making about menstrual management options. Existing studies that broadly discuss menstrual management of adolescents with developmental disabilities mention that parents/guardians sought premenarcheal anticipatory guidance. Common perimenstrual concerns among parents/guardians included menstruation hygiene and exacerbation of preexisting conditions.^18–19^

Females with epilepsy without ID also have concerns about initiating contraception, according to previous literature. Participants in these previous studies consistently identified contraception-drug interactions as a major factor in their decision-making process and noted that it is diﬃcult to make informed decisions about contraception without information about drug interactions.^12,20^ Our study mirrors these findings, as parents/guardians expressed substantial concerns about drug interactions.^3,5,8–10^ However, for females with epilepsy without ID, the teratogenic nature of certain antiseizure medications was a key factor in decision-making surrounding contraception.^20,21^ However, in our study, parents/guardians did not prioritize pregnancy prevention as a reason for starting contraception.

Our findings relate to previous studies that more broadly looked at how people with chronic disease make decisions about contraception. This literature largely pertains to adult women making independent decisions about contraception for pregnancy prevention.^20–23^ Participants in these studies expressed some concerns similar to those of the parents/caregivers in our study about the possibility that contraceptives could lead to worsening of disease symptoms or intolerable adverse effects.^22–25^ Participants in previous studies also expressed concerns about longer-acting methods that they could not easily reverse themselves.^25^ However, the risk-benefit calculation was often different given that most participants in our study were using contraception for menstrual concerns rather than pregnancy prevention, with some parents opting not to use contraception due to perceptions that risks outweigh benefits. Our study also builds on the previous literature by specifying adverse effects of high concern to these parents/caregivers, some of which were epilepsy-specific (ie, increased seizures, drug interactions with antiseizure medications) and some of which were not (ie, bone health, weight gain).^26^ The literature mentions the Nexplanon device being placed in alternative sites, such as the back, to help with medical trauma that can be associated with these procedures.^27^ Counseling about this option, along with more consistently offering intrauterine device insertion with anesthesia, can address parental concerns about increased medicalization for those with chronic disease.

There are several limitations of this study. One limitation is that we included only parents/guardians who could speak English proficiently. Therefore, the findings may not be transferable across different linguistic groups. Similarly, female caregivers were represented more than male ones in the study; the findings may not be generalizable to male caregivers. Another limitation is that this study did not address the newest option of subcutaneous depot medroxyprogesterone acetate. This newer option of contraception improves ease of use and gives a more convenient option to administer at home. Furthermore, qualitative research by nature aims to explore a topic in depth and elicit themes and therefore does not produce quantitative data.^28^ Future directions include conducting interviews with verbal people with epilepsy and mild-moderate ID and interviewing parents and patients with epilepsy without ID about their experiences surrounding contraception decision-making. Our research group is actively conducting the project involving verbal people with epilepsy and mild-moderate ID.

The findings of our study, in combination with the present literature, can help map out important discussion points and create clinical guidelines for providers to navigate SRH discussions and improve the quality of contraceptive counseling and care provided to individuals with ID and epilepsy. Counseling tips are outlined in Table 3 and can be used in combination with the present material.^14,29–40^

## Conclusion

In conclusion, our findings suggest that parents/guardians of AYAs with epilepsy and ID consider multiple factors when making decisions surrounding conraception. Clinicians can use the study findings to provide parents with information about contraceptive options and engage with parents’ goals, questions, and concerns regarding contraception. The study findings may inform interventions to improve contraceptive care for AYAs with epilepsy and ID, including development of discussion guides and decision aids for parents/caregivers.

## Figures and Tables

**Table 1:** Participant Demographic Characteristics

Parent age	Relationship to patient	Parent race/ethnicity	Parent gender Male (M), Female (F)	Patient race/ethnicity	Patient age	Developmental diagnosis	Verbal vs nonverbal	Medically intractable epilepsy	Current scheduled anti-seizure medications	Other therapies
69	Grandfather (guardian)	White	M	White	16	ASD	Verbal	yes	Lamotrigine, Clobazam	N/A
37	Mother (guardian)	White	F	White	18	CP, ID	Non-Verbal	yes	Topiramate, Diazepam	N/A
53	Mother (guardian)	White	F	White	13	Rett Syndrome	Non-Verbal	yes	Levetiracetam, Zonisamide	N/A
44	Mother (guardian)	White	F	White	18	CP, ID	Minimally Verbal	no	Levetiracetam, Valproic Acid	N/A
60	Mother (guardian)	White	F	White	25	Rett Syndrome	Non-Verbal	yes	Cannabidiol, Cenobamate	VNS
61	Mother (guardian)	White	F	White	25	ASD	Minimally Verbal	no	Topiramate, Diazepam	N/A
32	Mother (guardian)	White	F	White	13	ID	Verbal	no	Lamotrigine	N/A
46	Mother (guardian)	White	F	White	16	PDD	Non-Verbal	yes	Clobazam	N/A
42	Mother (guardian)	White	F	White	22	ASD, ID	Verbal	yes	Lamotrigine, Phenytoin	VNS
56	Mother (guardian)	White	F	White	22	ASD, ID	Verbal	yes	Lacosamide	VNS
56	Mother (guardian)	White	F	White	12	ID, Global Brain Injury	Non-Verbal	yes	Topiramate, Clonazepam	N/A
52	Mother (guardian)	White	F	White	28	LaFora Disease, ID	Verbal	yes	Rufinamide, Clonazepam, Valproic Acid, Perampanel, Levetiracetam, Zonisamde	N/A
46	Mother (guardian)	White	F	White	16	Rett Syndrome, ID	Non-Verbal	yes	Lamotrigine	N/A
48	Mother (guardian)	White	F	White	18	ID, CP	Verbal	no	Levetiracetam, Oxcarbazepine	N/A
42	Mother (guardian)	White	F	White	15	Hemimegalencephaly, ID	Non-Verbal	yes	Clobazam, Oxcarbazepine Levetiracetam, Gabapentin	N/A
55	Mother (guardian)	White	F	White	23	ASD, ID	Verbal	yes	Oxcarbazepine	N/A
50	Mother (guardian)	White	F	White	15	ASD, ID	Verbal	no	Lamotrigine, Zonisamide	N/A
52	Mother (guardian)	White	F	White	14	ID	Verbal	no	Levetiracetam	N/A
74	Mother (guardian)	White	F	White	19	ID, brain malformation	Verbal	no	Levetiracetam	N/A
46	Mother (guardian)	African American	F	African American	20	ID	Verbal	no	Levetiracetam, Carbamazepine	N/A
51	Mother (guardian)	Hispanic	F	Hispanic	21	Cornelia de Lange	Verbal	no	Lacosamide	N/A
45	Mother (guardian)	African American	F	African American	19	ID	Verbal	yes	Topiramate, Clobazam, Brivaracetam, Phenobarbital	N/A
46	Mother (guardian)	African American	F	African American	21	CP, ID	Non-Verbal	no	Diazepam	N/A
47	Sister (guardian)	African American	F	African American	18	ASD, ID	Non-Verbal	yes	Levetiracetam, Zonisamide, Clorazepate	N/A
57	Mother (guardian)	African American	F	African American	21	ID	Non-Verbal	yes	Valproic acid, Lamotrigine, Clobazam	VNS

ASD, autism spectrum disorder; CP, cerebral palsy; ID, intellectual disability.

**Table 2:** Key Themes and Quotes on the Preferences and Experiences of Parents/Guardians of Adolescents and Young Adults Regarding Decision-Making Surrounding Contraception

Theme	Representative quotations
**1. Parents’/caregivers’ desire for birth control for their child was more centered around menstruation-related concerns rather than pregnancy prevention.**	*“I expressed my concerns because she was in so much pain with it and initially we were going to do three periods a year and and I had expressed, you know, my concern about the increased seizure activity with her period. But then when we she had one that May it was bad and actually myself and when we had another I actually emailed and asked her, can we just do continuous so she doesn’t get one at all.”* *“So she would have the pain, the cramping, in addition to having the emotional swings, the anger, all those kinds of things.”* *“But [she] would never get pregnant. My concerns are, is if she was in a facility,* *if there was ever, unfortunately, a sexual assault, she could become pregnant.”*
**2. Many parents were afraid of potential adverse effects of contraception for their children, including perceptions that risks might outweigh benefits.**	*“That’s always been my concern about giving her anything other than the seizure meds due to interaction. So that would have been my number one concern, making sure that it did not interact.”* *“I don’t know if she’s got a pain from a side effect because she can’t tell me that.”* *“…. my primary concerns is just the weight gain and then how it’s going to affect her bones.”*
**3. A common important factor in contraceptive decision-making was ease of administration, and, generally, the pill form was preferred.**	*“And I really didn’t want to do birth control pills like [my daughter] has over the years taken so many meds.”* *“My concern with her if she had a IUD. Oh, yeah. If it were like if it was not like if it was coming out or if it was causing her pain or I figured the pill was the easiest route to go.”* *“And [she] would have no way of telling me if it was hurting her or if it, you know, if it moves or until something drastic would happen.”*
**4. Longer-acting methods, if chosen, were largely selected due to trusting recommendations from health care providers yet were often feared due to perceptions of lower ease of reversibility if problems arise, as well as greater challenges in delivery or placement.**	*“It was because if like the shot wasn’t the right doses, it still was in her body and the pill. We could adjust and change, like kind of right away.”* “Our fear was that once she has the shot, obviously, if something would not go right. You know, you can’t reverse that. And then it’s done. You know, you can’t change.”

**Table 3:** Evidence-Based Counseling Tips

Topic	Key points
Bone density^29–30^	• LARCs do not affect peak bone mass acquisition or future fracture risk • Adverse effects of DMPA on bone health are likely reversible once medication is discontinued • Routine DXA scans are not recommended for adolescents on DMPA • Contraceptive patch and vaginal ring do not adversely affect bone health in adults, but limited data in adolescents • Combined oral contraception use in adults is not associated with increased fracture risk
Weight management^31–39^	• Nonhormonal IUDs are not associated with weight fluctuations • For patients who are more sensitive to weight changes, counseling on the possibility of weight gain is important
Antiseizure medication interactions with estrogen & progesterone contraceptives^14 , 41–42^	• Many ASMs induce cytochrome P450 enzymes → causing lower concentrations of estrogen and progesterone → potentially increasing risk of contraceptive failure • Using lamotrigine with exogenous estrogen can significantly reduce lamotrigine serum concentrations → potentially increasing risks of seizures • No interactions between IUDs and ASMs • For a comprehensive account of drug interactions between ASMs and contraceptives please refer to Tables 1 and 2 from Weatherspoon et al^36^

ASMs, antiseizure medications; BMI, body mass index; DMPA, depot medroxyprogesterone; DXA, dual-energy x-ray absorptiometry; IUD, intrauterine device; OCPs, oral contraceptives.
